# *Leishmania infantum* UBC1 in Metacyclic Promastigotes from *Phlebotomus perniciosus*, a Vaccine Candidate for Zoonotic Visceral Leishmaniasis

**DOI:** 10.3390/vaccines10020231

**Published:** 2022-02-03

**Authors:** Jaime Larraga, Pedro J. Alcolea, Ana M. Alonso, Luis T. C. Martins, Inmaculada Moreno, Mercedes Domínguez, Vicente Larraga

**Affiliations:** 1 Departamento de Biología Molecular y Celular, Centro de Investigaciones Biológicas Margarita Salas (Consejo Superior de Investigaciones Científicas), 28040 Madrid, Spain; jlarraga@cib.csic.es (J.L.); pjalcolea@cib.csic.es (P.J.A.); amalonso@cib.csic.es (A.M.A.); luistccm@gmail.com (L.T.C.M.); 2 Unidad de Inmunología, Centro Nacional de Microbiología, Virología e Inmunología Sanitarias (Instituto de Salud Carlos III), 28220 Majadahonda, Spain; imoreno@isciii.es (I.M.); mdominguez@isciii.es (M.D.)

**Keywords:** *Leishmania*, vaccines, ubiquitin-conjugating enzyme E2, infection

## Abstract

*Leishmania* parasites cause outstanding levels of morbidity and mortality in many developing countries in tropical and subtropical regions. Numerous gene expression profiling studies have been performed comparing different *Leishmania* species’ life-cycles and stage forms in regard to their distinct infective ability. Based on expression patterns, homology to human orthologues, in silico HLA-binding predictions, and annotated functions, we were able to select several vaccine candidates which are currently under study. One of these candidates is the *Leishmania infantum* ubiquitin-conjugating enzyme E2 (LiUBC1), whose relative levels, subcellular location, in vitro infectivity in the U937 myeloid human cell model, and protection levels in Syrian hamsters against *L. infantum* infection were studied herein. LiUBC1 displays a low level of similarity with the mammalian orthologs and relevant structure differences, such as the C-terminal domain, which is absent in the human ortholog. LiUBC1 is present in highly infective promastigotes. Knock-in parasites overexpressing the enzyme increased their infectivity, according to in vitro experiments. Syrian hamsters immunized with the recombinant LiUBC1 protein did not show any parasite burden in the spleen, unlike the infection control group. The IFN-γ transcript levels in splenocytes were significantly higher in the LiUBC1 immunized group. Therefore, LiUBC1 induced partial protection against *L. infantum* in the Syrian hamster model.

## 1. Introduction

Leishmaniasis is a disease caused by trypanosomatid parasites from the genus *Leishmania*. Human leishmaniasis is classified according to the clinical manifestations into cutaneous (CL), mucocutaneous (MCL), and visceral leishmaniasis (VL), as well as according to the species involved. VL caused by *Leishmania infantum* is a zoonosis (ZVL), whereas VL caused by *L. donovani* is anthroponotic. In both cases, VL is fatal without treatment, with about 50,000 annual deaths [1]. HIV–*Leishmania* coinfections have been reported [2,3,4,5,6]. Canids are described as *L. infantum* (syn. *L. chagasi*) reservoirs in the Northern Mediterranean basin, Central Asia, and South America [7,8]. The vector in the Mediterranean basin is *Phlebotomus perniciosus* (Psychodidae: Phlebotominae). A human *L. infantum* outbreak declared more than a decade ago in central Spain is still active [9,10,11,12]. In contrast to human VL, dogs display variable cutaneous and visceral clinical signs which appear simultaneously. There are also variable profiles for the clinical signs that range from asymptomatic to severe. These include onicogriposis, weight loss, asthenia, cutaneous lesions, conjunctivitis, anorexia, lymphadenopathy, and hepatic splenomegaly [13,14]. *Leishmania* spp. parasites have a life cycle defined by two stages. The promastigotes are fusiform motile cells with a flagellum emerging from the cellular body anterior pole. This stage differentiates into the procyclic form in the sand fly gut, with low infective ability; this form then migrates towards the anterior part of the gut, transforming into a highly infective form called metacyclic. These promastigotes are inoculated in the mammalian host’s dermis during blood meal intake. The promastigotes are mainly cleared by the complement system; those resistant to this action are internalized by phagocytic mononuclear cells, where they differentiate into the obligate intracellular amastigote stage and multiply. The amastigotes are released when the host cell collapses and infect other phagocytes. The sand fly be-comes infected by feeding from an infected mammal’s blood, completing the parasite cycle [15].

The treatment of this disease is far from being solved. The available drugs, such as pentavalent antimonials, mitelfosine, and amphotericin B, are the first line of treatment. As a consequence of the prolonged use of these drugs, resistant parasites have emerged, resulting in clinical relapses. Another disadvantage are the side effects [16,17]. Therefore, vaccines are desirable to prevent *Leishmania* infections, but no vaccine against human leishmaniasis has been developed. All vaccines available against leishmaniasis confer only partial protection [18,19,20,21]. We have developed the pPAL-LACK vaccine with protection levels of approximately 60% against canine leishmaniasis [22]. This vaccine is in the final stages of evaluation by the European Medicines Agency (EMA). Its combination with other antigens may improve its efficacy. *Leishmania*-hamster is an appropriate infection model for VL [23,24]. However, the immune response has not been characterized due to the lack of available reagents for immunological studies [25].

The immune response against *Leishmania* spp. is partially understood, though only in mice with the *L. major* infection model. In this case, T helper type 1 lymphocyte response (Th1)-producing interferon-γ (IFN-γ) leads to protection, whereas Th2 response leads to susceptibility [26]. In the case of the canine host, this response is more complex, with an initial increase in both Th1 and Th2 subpopulations [27]. The mechanisms responsible for counteracting the action of macrophages may be present at the promastigote stage before the infection process by the overexpression of genes responsible for encoding proteins involved in resistance mechanisms, or by the involvement of post-translational modifications of proteins participating in the resistance mechanisms. Most of the *L. infantum* genes are constitutively expressed throughout the differentiation processes of the parasite, and less than 5% are differentially expressed [28]. However, proteomic studies have shown that up to 18% of the proteins appear differentially expressed among the distinct parasite stages [29]. Additionally, there is a low correlation between mRNA and protein levels [30]. Thus, both post-transcriptional and post-translational control may play an important role in the regulation of protein expression in *Leishmania* spp. 

Previous studies in our laboratory showed the overexpression of the LinJ.33.2910 gene (www.tritrypdb.org) encoding the E2 ubiquitin-conjugating enzyme LiUBC1 in infective metacyclic promastigotes obtained from the *P. perniciosus* vector midgut [31,32]. This stage may be ideal for acquiring vaccine candidates. The expression profile, HLA-binding predictions, and sequence dissimilarity with mammalian orthologs suggest that this molecule may be a vaccine candidate [33].

Ubiquitin is a polypeptide that binds covalently to substrate proteins and either modifies the protein function or induces its degradation by the cell’s proteasome machinery. The conjugation of ubiquitin to substrate proteins has been shown to have an essential role in controlling many cellular processes, including cell division, DNA repair, and protein synthesis [34]. The ubiquitin proteasome system is one of the main post-translational regulation mechanisms in eukaryotic cells [35]. In fact, the role of this type of protein in modifying the ubiquitination process to improve viral replication in infected cells has been described [36]. During the protein ubiquitination process, the ubiquitin-activating enzyme (E1) activates the carboxy terminus of ubiquitin. The activated ubiquitin is then transferred from E1 to the ubiquitin-conjugating enzyme (E2). Finally, the ubiquitin ligase enzyme (E3) catalyzes the transfer of ubiquitin from E2 to lysine amino acid residues in the substrate protein [37]. There is growing evidence for the possible relation between the downregulation of this system and the appearance of diseases related to the erroneous folding of proteins, mainly neurodegenerative diseases, such as Lewy bodies in Parkinson’s disease, which display an increase in ubiquitin bound to aggregated proteins [38,39]. Despite these suggested functions, little is known about their role in cellular physiological mechanisms. Therefore, the study of ubiquitin-related proteins deserves more attention, and lower eukaryotes should provide valuable information. In fact, this protein seems to be related to the clearance of defective proteins from the cell nucleus in *Saccharomyces cerevisiae* [40]. Parasitic protozoa are complex unicellular organisms that undergo distinct modifications to adapt themselves to the different physicochemical environments corresponding to their distinct hosts during their biological cycles and, therefore, may be a useful model to study these regulation processes. One of the main features of *Leishmania* parasites are the morphological changes they undergo from the insect vector midgut to the interior of the phagocyte vacuoles in the mammalian host. These changes in shape and size require the cytoskeleton to adapt and strictly regulate the proteolysis mechanisms [41]. In fact, several processes regulated by the proteasome complex have been described in *Leishmania* spp. In *L. donovani*, it has been noted that the key enzyme methionine adenosyl transferase is negatively regulated by the proteasome complex. In the same way, ubiquitination has been associated with mRNA regulation in *L. major* [42] or with resistance to antimonial treatment in *L. tropica* [43]. 

The present work describes the structural modeling, relative levels, subcellular localization, and biological role in infectivity of LiUBC1, and presents evidence supporting the conclusion that LiUBC1 is a vaccine candidate that protects Syrian hamsters against *L. infantum* infection. 

## 2. Materials and Methods

### 2.1. Parasites and Whole Protein Extracts

*L. infantum* promastigotes of the IPER/ES/2013/ATE1FL6 isolate were obtained from the stomodeal valve of *P. perniciosus* specimens captured during the Fuenlabrada human outbreak [9]. Promastigotes were cultured at 26 °C in Novy–Nicolle–McNeal (NNN) medium. In all cases, promastigotes were transferred to complete medium (CM) composed of RPMI 1640 supplemented with 2 mM glutamine (Gibco BRL, Waltham, MA, USA), 10% heat-inactivated (56 °C, 1 h) fetal bovine serum (Cambrex, East Rutherford, NJ, USA), 100 μg of streptomycin, and 100 IU penicillin/mL (Gibco BRL). Parasites were grown at 28 °C. Culture samples were taken every 24 h. 

At each experimental time point, 2 × 10^8^ promastigotes were centrifuged at 200× *g* for 5 min and washed three times with PBS. The sediment was resuspended in 100 µL of lysis buffer (8.4 M urea, 2.4 M thiourea, 4% CHAPS *p*/*v*, 50 mM DTT, 1% Triton X-100, one tablet of protease inhibitor cocktail (Roche, Basel, Switzerland), and 1 unit of DNAse (New England Biolabs, Ipswich, MA, USA)). After gently shaking for 20 min, the mixture was centrifuged at 8000× *g* for 5 min at 4 °C. The supernatant was recovered and stored at −80 °C before use.

Peanut lectin nonagglutinating (PNA) promastigotes were obtained in stationary phase. First, they were centrifuged at 2000× *g* for 10 min in CM at a cell density of 2 × 10^8^ cells/mL and incubated with 50 µg/mL of PNA (Sigma-Aldrich, St. Louis, MO, USA) at room temperature (RT) for 30 min. Agglutination and sedimentation was checked with light microscopy. The pellet obtained was resuspended in 1 mL of the remaining supernatant and taken to the initial volume of 10 mL with CM at the same concentration of PNA and centrifuged at 200× *g* for 10 min. The centrifugation step was repeated to obtain the PNA+ promastigotes. The last supernatant was centrifuged at 2000× *g* to obtain the PNA- promastigotes. The whole process was checked by light microscopy.

### 2.2. Ethics 

All possible animal welfare measures were applied to 14 *Mesocricetus auratus* specimens required for the challenge experiment, such as optimal temperature, ad libitum feeding, refinement, disease follow-up, and environmental enrichment. The experimental design, the procedures, and the safety measures were approved by the Instituto de Salud Carlos III Ethical Advisory Committee for Animal Experimentation following the EU Directive 2010/63 and the Spanish regulation RD53/2013. Manipulation of rabbits was also carried out according to the animal welfare rules and supervised by the Consejo Superior de Investigaciones Científicas (CSIC) Ethical Committee (EU Directive 2010/63 and Spanish regulation RD53/2013).

### 2.3. LiUBC1 Expression and Purification

The LiUBC1 gene CDS, including an upstream 6xHis-tag coding sequence was PCR-amplified (BamHI-LiUBC1-Fw 5′-TAAGGATCCCCTCTACGGCGGCT-3′ and BamHI-LiUBC1-Rv 5′-CAAAAGCTTTCAGATGCGGCGCAGC-3′) and cloned in the pQE30 expression vector using XL1-blue chemically competent cells as the recipient *E. coli* strain. *E. coli* M15 cells were transformed with pQE30-LiUBC1 and a 10 mL preinoculum grown at 37 °C in LB medium containing ampicillin (100 µg/mL) and kanamycin (50 µg/mL). A 0,5 L flask was seeded with 5 mL of inoculum and grown to an OD_595_ value of 0.5. Heterologous expression was then induced by adding 1 mM IPTG and incubating for 4 h at 37 °C. Thereafter, the bacterial cells were centrifuged at 6000× *g* for 15 min at 4 °C and washed with 0.9% NaCl solution. Per gram of pellet, 10 mL of lysis buffer (1 M KCl, 60 mM imidazole, 1% Brij 58, 10% glycerol, and 1 mM benzamidine) was added. The lysate was stored at −80 °C until usage. Once thawed, it was centrifuged at 20,000× *g* for 1 h at 4 °C. LiUBC1 contains the 6xHis tag in the N-terminus and was found mainly in the pellet, which was resuspended in 3 mL of 5 M GuHCl for subsequent purification in denaturing conditions. After centrifugation, the extract was subject to affinity chromatography in a 6xHis-loaded HiTrap FF 1 mL column (GE Healthcare, Little Chalfon, UK) using an FPLC ÄKTA system (GE Healthcare, Little Chalfon, UK). After washing with increasing concentrations of imidazole, 6xHis–LiUBC1 elution was performed with 700 mM imidazole and 5 M GuHCl. The purified protein fractions were analyzed by SDS-PAGE. 

### 2.4. Antibody Obtainment

The specific anti-LiUBC1 polyclonal antibody was obtained using New Zealand rabbits, each inoculated with four doses of 500 µg of the purified LiUBC1 protein in 0.5 mL PBS buffer. The LiUBC1 protein was obtained by excision of the purified band and subsequent gel diffusion after SDS-PAGE using prestained TGX gels (BioRad^®^, Hercules, CA, USA). The initial dose was emulsified with Freund’s complete adjuvant (1:1) and the following three doses with incomplete Freund’s adjuvant, each inoculated at three-week intervals. 

### 2.5. Protein Expression Level Determination by Western Blot

Relative expression levels of proteins between different cellular stages were carried out by Western blot experiments, according to a previously described protocol [44]. In short, previously obtained extracts from different days of promastigote culture as well as amastigote-like cells were quantified by the Bradford method [45], and 20 µg of each sample was run through SDS-PAGE [46] and transferred to nitrocellulose membranes (0.45 µm, BioRad labs). LiUBC1 was detected using the specific polyclonal antibody generated (1:100) and HRP-conjugated goat antirabbit IgG (1:2000) as the secondary antibody (Dako, Agilent Technologies, Santa Clara, CA, USA). A primary antibody against gGAPDH (1:10000) was used for the internal standard [47]. Detection was carried out by using an ECL Western Blotting Detection Kit (GE Healthcare) and a LAS-3000 Image Reader (GE Healthcare).

### 2.6. Generation of L. infantum LiUBC1 Knock-In Promastigote Line

The obtainment of an *L. infantum* cell line overexpressing LiUBC1 was carried out with the vector pIRmcs3 [48]. The LinJ.33.2910 gene was initially cloned in the DH5α *E. coli* strain. *L. infantum* promastigotes were washed in electroporation buffer (132 mM NaCl, 8 mM KCl, 8 mM Na_2_HPO_4_, 1.5 mM KH_2_PO_4_, 0.5 mM potassium acetate, 90µM calcium acetate, pH 7.0), sterilized by filtration (0.22 µm), and resuspended in 400 µL of the same buffer to a final concentration of 7 × 10^6^ cells/mL. The electric pulse (1 KV/24 Ω/28 ms) was delivered in the presence of the linearized plasmid pIRmcs3 containing the LiUBC1 gene (3 µg DNA). The same conditions were used with the pIRmcs3 plasmid without insert for the obtainment of a control promastigote line. During the initial 24 h of culture, transfected promastigotes and controls were resuspended in CM supplemented with 20% HIFBS and 10% stationary promastigote culture medium sterilized by filtration. After 24 h, 100 µg/mL of the selection agent nourseothricin (Jena Bioscience, Jena, Germany) was added to the cultures. After one week, the amount of nourseothricin was increased to 120 µg/mL and transfected promastigotes were cultured in the presence of the antibiotic. In all experiments, promastigotes transfected with the pIRmcs3 plasmid without the LinJ.33.2910 gene were used as controls.

### 2.7. LiUBC1 Cellular Detection by Indirect Immunofluorescence (IIF)

*L. infantum* promastigotes (2 × 10^5^) were sedimented onto a 0.2 cm^2^ area on a glass slide and fixed with 4% paraformaldehyde solution for 5 min at RT. After three washes with PBS in a Coplin jar for 5 min, *L. infantum* promastigotes were permeabilized with a 0.5% Triton X-100 solution in PBS for 5 min. Blocking was carried out with a 0.1% solution of Tween-20 in PBS containing 5% *w/v* skimmed milk in a wet chamber. Promastigotes were incubated with specific polyclonal antibody anti-LiUBC1 in blocking buffer (1:50) for 1 h. After a single wash, cells were incubated with the secondary antibody antirabbit IgG conjugated to the fluorophore Alexa Fluor 488 (Jackson Immunoresearch, West Grove, PA, USA) (1:200) in blocking buffer for 1 h in the dark. Ten minutes before the end of this incubation, 10 µL of 10 µg/mL DAPI (Thermo Fisher Scientific, Waltham, MA, USA) in PBS was added. The cells were finally washed three times with PBS in the dark and preparations were mounted with Mowiol 4-88 (Sigma-Aldrich, Burlington, MA, USA). Images were obtained in a Spectrum Laser confocal Leica TCS-SP5-OABS microscope previously adjusted to the negative control (preimmune serum 1:50).

### 2.8. Transmission Electron Microscopy (TEM) Detection of LiUBC1

Promastigotes were fixed with 2% *p*-formaldehyde and 0.2% glutaraldehyde in 0.1 M sodium kakodylate, 4.5% sucrose, pH 7.4, for 30 min at 4 °C. The samples were dehydrated in an ethanol–ethyleneglycol sequential mixture (50–100%) in a temperature range of 0°C to −35 °C at 30 min intervals. Then, promastigotes were transferred to a Lowycril K4M solution in ethanol (1:1 and 1:2) and finally to pure Lowicryl K4M overnight. Subsequently, the samples were introduced into gelatin capsules and polymerized by UV light for 72 h and then cut with an ultramicrotome. 

The promastigote preparations were incubated with the anti-LiUBC1 antibody (see Section 2.4) for 60 min at 37 °C. After four washes of 5 min, the samples were incubated with antirabbit IgG secondary antibody conjugated with colloidal gold (1:10). After three additional washes with buffer containing 0.1% Triton X-100 in milliQ water, the samples were incubated with 1% uranyle acetate in bidistilled water for 10 min, washed twice with water for 1 min, mounted, and observed with a JEOL JEM-1230 electron microscope.

### 2.9. Mass Spectrometry LC–MS/MS Analysis

LiUBC1 samples were dissolved in Laemmli sample buffer and run through a 10% SDS-PAGE gel. Protein bands were excised from the gel and digested in-gel with trypsin, as described in [49,50]. Peptides were separated with an Easy-nLC 1000 Nano System (Thermo Fisher Scientific, Waltham, MA, USA) using a precolumn Acclaim PepMap 100 (Thermo Fisher Scientific) and an RSLC PepMap C18, 15 cm long, 75 µm inner diameter, and 3 µm particle size column (Thermo Fisher Scientific). The flow rate was 300 nL/min using 0.1% formic acid in water (FAW) and 0.1% formic acid in 100% acetonitrile (FAA). The gradient profile was set as follows: 0–35% FAA for 90 min, 35–100% FAA for 4 min, 100% FAA for 8 min. The sample input was 4 µL.

A Q Exactive LC–MS/MS system (Thermo Fisher Scientific) was used to perform the MS analysis. Ionization was set at a liquid junction voltage of 1800 V and a capillary temperature of 270 °C. The software scan method was set with *m*/*z* values equal to 400–1500, a resolution value of 70,000 for the Orbitrap analyzer (for *m*/*z* 200), target automatic gain control (AGC) equal to 3 × 10^6^, and an injection time up to 100 ms. The 10 most intense precursor ions were chosen for MS/MS fragmentation, which was performed with a normalized collision energy of 27 eV and the following settings applied for acquisition: starting *m*/*z* value of 100; AGC target of 2 × 10^5^ with a 8 × 10^3^ threshold; 17,500 resolution (*m*/*z* 200); 2 *m*/*z* isolation window; and 100 ms maximum IT. The ions that were unassigned, singly charged, or bore ≥7 protonations were excluded from subsequent analysis, applying a dynamic exclusion time of 20 s to discriminate previously selected ions.

Whole protein samples were obtained on days 1, 3, and 5 of growth using a lysis buffer containing 8.4 M urea, 2.4 M thiourea, 4% *w/v* CHAPS, 50 mM DTT, 1% Triton X-100, one tablet of a protease inhibitor cocktail (Roche), and 50 µg/mL benzonase (Merck, Kenilworth, NJ, USA). The protein samples were precipitated with methanol:chloroform:water (4:1:3), resuspended in 50 mM triethyl ammonium bicarbonate (TEAB), and labelled using an 8-Plex iTRAQ kit (AB SCIEX) according to manufacturer instructions. The labelled samples were grouped and purified using MCX cartridges (Waters) to remove excess iTRAQ reagent and nonpeptidic residual materials. Peptides were eluted with 1 mL of methanol:ammonium hydroxide (9:1), dried, and fractionated by liquid chromatography. After desalting with C18 OMIX (Agilent Technologies), samples were kept at −80 °C until their LC–MS/MS analysis. All peptide separation was carried out using an Easy-nLC 100 (Thermo Fisher Scientific) system. The elution stream was 300 nL/min using formic acid in acetonitrile from 0.1% to 100%. MS analysis was conducted using an OrbiTrap Mass Spectrometer (Thermo Fisher Scientific). For quantification, labeled peptide mixtures were reconstituted in formic acid 0.1%, trapped in a precolumn Acclaim PepMap 100 (Thermo Fisher Scientific), and eluted in an Acclaim PepMap C18 column. Each analysis was carried out in duplicate. 

Proteome Discoverer software (version1.4.1.14) (Thermo Fisher Scientific) was used for iTRAQ analysis. The *.raw files containing the mass spectra were analyzed with SEQUEST against the reference *L. infantum* JPCM5 genome, which contains 8062 protein entries. Mass tolerance was adjusted to 10 ppm for precursors and 0.02 Da for fragments. Other settings were 2 missed cleavages, carbamidomethylation of cysteines (fixed), methionine oxidation, and N-terminal acetylation (variable). The Percolator algorithm 9 [51] was used to filter identified peptides, setting 0.01 as q-value threshold.

### 2.10. L. infantum In Vitro Infection Experiments

The U937 cells (CRL-1593.2^TM^, ATCC^®^, Manassas, Virginia, USA) were maintained in Roux flasks at 37 °C in the presence of a 5% CO2 atmosphere. On an 8-well cell culture slide, 150 µL of cell suspension per well was fixed at a cell density of 2 × 10^8^/cm^2^. Cells were stimulated with 20 ng/mL of phorbol myristate acetate (PMA) for 72 h and infections were carried out by adding wild-type and LiUBC1-transfected *L. infantum* promastigotes to the cell cultures at a promastigote:U937 cell ratio of 10:1 for 2 h. After infection, cells were washed with CM. All the biological replicates were carried out in triplicate. After 24 or 48 h, the culture slides were washed and 150 µL of fixing solution (ethanol:acetic acid (3:1)) was added to each well. Wells were removed and the slides were stained with Diff-Quik solution (Dade Behring, Deerfield, IL, USA). The percentage of infected cells as well as the number of amastigotes per cell were calculated in all biological replicates by using the Student’s t-test implemented in Sigmaplot 11 software (Sigmaplot^®^, San Jose, CA, USA).

### 2.11. Animal Challenge Experiments

Of a total of 14 animals (*M. auratus*), 6 were used as controls and 8 were inoculated intraperitoneally with 20 µg of recombinant LiUBC1 protein in 1 mL of PBS. Controls were inoculated with PBS. The second inoculation was given three weeks later. After two weeks, animals were challenged with 10^7^ *L. infantum* metacyclic promastigotes (MCAN/ES98/10445) in 200 µL of PBS. The animals were euthanized according to the EU rules for animal welfare (EU Directive 2010/63 and Spanish regulation RD52/2013), and spleen and blood were obtained for analysis.

### 2.12. Parasite Burden Evaluation and Immune IgG Response Determination in M. auratus Model

At the end of the challenge experiments with *M. auratus*, the spleens were extracted aseptically, weighed, homogenized, and resuspended in 3 mL of Schneider´s Drosophila medium supplemented with 10% HIFBS (HyClone, Thermo Fisher Scientific), penicillin (100 U/mL), streptomycin (50 mg/mL, Lonza^®^), 20 mM HEPES (Sigma-Aldrich), and 1% sterile urine. Four samples of the homogenized suspension were seeded and diluted six times in a 96-well plate. Plates were incubated for two weeks and parasites were detected in the well cultures by optical microscopy (200×). Parasite burden was calculated as the geometric mean of the parasites detected at the maximum dilution described [52].

The blood sample was allowed to clot at RT for 20 min and was centrifuged at 10,000× *g* at room temperature for 10 min. The serum was stored at −80 °C and used later for a total IgG antibody determination against total *L. infantum* soluble antigen (SLA) by ELISA. First, a flat bottom plate was lined with 100 µL of a 10 µg/mL SLA solution in 3.36 mM carbonate–10 mM bicarbonate buffer overnight at 4 °C. Subsequently, it was blocked with a 1% BSA solution in PBS and washed three times with a 0.1% BSA and 0.03% Tween 20 solution in PBS. Next, 100 µL of a 1:20 serum sample was added to each well and left for 30 min at RT. Meanwhile, protein A (Sigma-Aldrich, Burlington, MA, United States) was prepared at a concentration of 0.19 µg/mL using a wash solution. The samples were washed before and after incubation, which was performed with 100 µL of the protein A solution for 2 h at RT. The last wash was carried out with PBS. An o-phenylenediamine dihydrochloride (OPD) tablet was dissolved in 12 mL of citrate buffer (5.2 mM citric acid, 12.7 mM Na_2_HPO_4_, pH 5) and 15 µL H_2_O_2_. To each well was added 100 µL of said solution, and the colorimetric reaction was allowed to develop for 20 min. The reaction was stopped by adding 50 µL of a 1% SDS solution. The absorbance was read at 450 nm using a Microplate Reader 680 (BioRad^®^, Hercules, CA, United States) and Microplate Manager 5.2.1 software (BioRad^®^).

### 2.13. Relative IFN-γ Expression Levels in Splenocytes by qRT-PCR

Whole RNA samples were obtained using TRIzol^®^ reagent (Life Technologies, Carlsbad, CA, USA). First-strand cDNA synthesis was performed starting from 10 μg of total RNA. First, the RNA was mixed with 6 μg of random hexamer primers (Life Technologies). The mixture was denatured at 70 °C for 2 min and immediately cooled on ice. Then, the samples were incubated at 46 °C for 3 h with 570 μM of each dNTP, 10 μM DTT, and 600 U SuperScript III Reverse Transcriptase (Life Technologies) in 30 μL of reaction volume. Next, RNA degradation was performed using 1U RNaseH (Life Technologies) at 37 °C for 30 min. Enzyme denaturation was performed at 65 °C for 10 min. The resulting cDNA samples were purified using QiaQuick PCR Purification Kit (Qiagen, Hilden, Germany). Syrian hamster IFN-γ TaqMan assay no. Cg04455412_m1 (primers and FAM-NFQ MGB probe, Applied Biosystems, Waltham, MA, USA) were mixed with cDNA samples (10, 2, and 0.4 ng cDNA per reaction set by serial dilutions) and with TaqMan Universal Master Mix 2X (Life Technologies) in 10 μL reaction volume. Thermal cycling runs were performed in a 7900HT Fast Real-Time PCR system. Thermal cycling conditions set in SDS 4.1. software (Life Technologies) were: 95 °C, 5 min; 40 × (95 °C, 30 sec; 60 °C, 1 min, data acquisition). The β-actin taqman assay no. Cg04424027_gH (Applied Biosystems) was used as the reference gene for quantification using the ΔΔCt [53]. The Mann–Whitney U test was applied to the 2^−^^ΔΔCt^ differential expression value at 0.01 significance level.

### 2.14. Sequence Alignment and Molecular Modeling

Protein sequences were retrieved from the NCBI database and the alignments were performed using the Clustal Omega software. 

The molecular model predictions were obtained with i-Tasser. The structures were taken from Protein Data Bank (PDB) and the final comparative analysis was performed using PyMol program. 

## 3. Results

### 3.1. Primary Structure of LiUBC1

**Figure 1:**
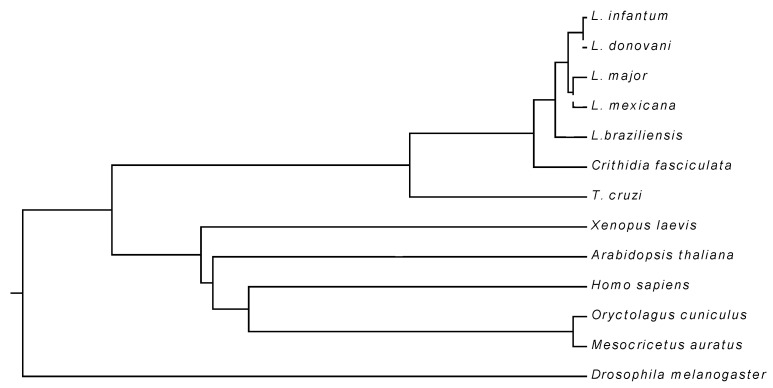
Phylogenetic analysis of the LiUBC1 protein: the phylogenetic tree with different species of the trypanosomatid family and with other orthologs; trypanosomatid orthologs are evolutionarily close, while mammalian orthologs are more distant.

The comparative analysis of the LiUBC1 sequence (encoded by the LinJ.33.2910 gene) with several ortholog proteins showed high identity (>80%) within the genus *Leishmania*, including *L. braziliensis*, a species from the *Viannia* subgenus. The UBC1 from *Trypanosoma cruzi* is evolutionarily more distant, as 50% sequence identity was observed. The similarity with mammalian orthologs is lower, with values ranging between 30% and 40% in the human, hamster, and rabbit orthologs. *Arabidopsis thaliana* showed an amino acid identity value in the same range as mammalians (Figure 1, Table 1)

The results indicated a high level of LiUBC1 conservation within the genus, in both the *Leishmania* and *Viannia* subgenera. Identity decreases to ~50% within the *Trypanosoma* genus and further in the higher eukaryotes, especially mammals. However, the role of the LiUBC1 orthologs is presumably maintained, because the N-terminal domain is the most conserved region between these species (Figure S1). Taking into account that percentages of identity only provide partial information about the tertiary or quaternary structure of the proteins, as well as about the final functional performance of the proteins, we determined the predicted structure of the enzyme based on known structures. 

### 3.2. Molecular Models of LiUBC1

We predicted the LiUBC1 model with solved structures of orthologs from *Homo sapiens* (1ZDN) [54] and *S. cerevisiae* (1TTE) [55], retrieved from PDB. 

**Figure 2:**
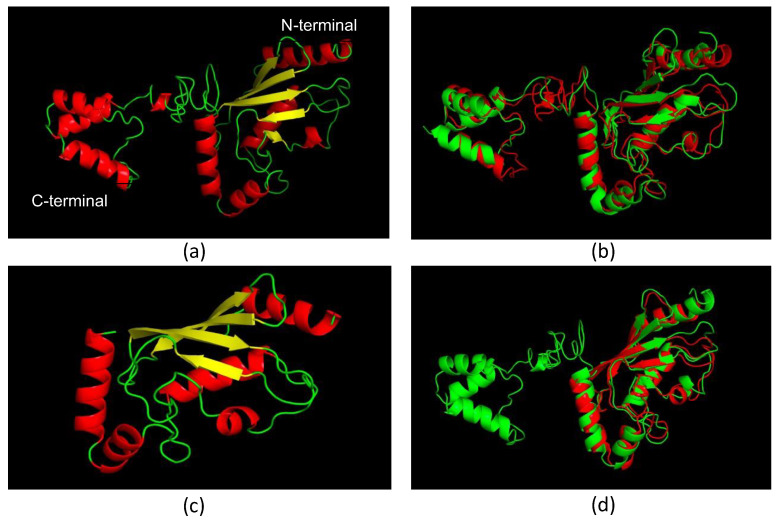
Structure modeling of LiUBC1 compared with resolved structures of homologous proteins: (**a**) LiUBC1 structure model indicating the C- and N-termini (α-helixes in red, β-sheets in yellow, random coil in green); (**b**) comparison of LiUBC1 (red) with the ortholog protein from *S. cerevisiae* (green) ScUBC1; (**c**) structure of the *H. sapiens* ortholog protein (HsUBC1) (α-helixes in red, β-sheets in yellow, random coil in green); (**d**) comparison of LiUBC1 (green) and HsUBC1 (red).

The predicted LiUBC1 structure is composed of a C-terminal domain and a UBC catalytic core. This structure is similar to the structure of the *S. cerevisiae* E2 ubiquitin-conjugating enzyme (Figure 2B), which has been described as one homodimer [53]. The human ortholog enzyme lacks the C-terminal domain (Figure 2C) present in LiUBC1 and *S. cerevisiae* proteins, but the N-terminal end, which contains the enzyme’s active center, also displays a very similar structure (Figure 2D). 

### 3.3. LiUBC1 Purification

**Figure 3:**
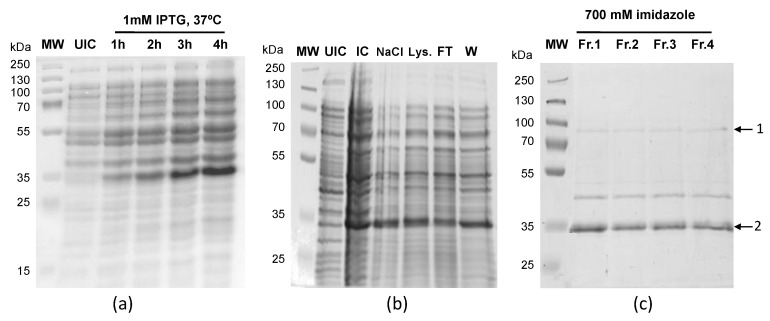
LiUBC1 expression and purification: (**a**) LiUBC1 protein expression detection by SDS-PAGE in the presence of 1 mM IPTG, induction from 1 to 4 h at 37 °C; (**b**) UIC—uninduced culture, IC—induced culture, NaCl—NaCl wash, Lys.—Lysate, FT—flow through, W—wash with 200 mM imidazole; (**c**) LiUBC1 eluted in 700 mM imidazole. Arrows indicate the LiUBC1 protein and possible aggregates.

The LinJ.33.2910 gene, which encodes LiUBC1, was cloned in the pQE30 expression vector in the *E. coli* XL1-blue strain, and the M15 strain for LiUBC1 overexpression in a heterologous system. LiUBC1 was expressed in the presence of 1 mM IPTG at an optimal temperature of 37 °C (Figure 3A). Most of the overexpressed protein was located in the nonsoluble fraction (Figure 3B). The LiUBC1 protein was solubilized with a buffer containing 5 M GuHCl and 40 mM imidazole and purified in batch using a Ni^2+^-NTA agarose column. Discontinuous elution was performed in three steps using 100, 400, and 700 mM imidazole, respectively. Most of the LiUBC1 protein was eluted at an imidazole concentration of 700 mM. The purified protein presented a complex pattern with bands that may correspond to the molecular weights of the LiUBC1 protein (26 kDa plus the histidine tag), as well as bands that may correspond to aggregates (Figure 3C).

To confirm the presence of LiUBC1 aggregates, the bands were excised, in-gel SDS-PAGE trypsin-digested, and analyzed by nLC–MS/MS (Table 2).

The two bands correspond to the LiUBC1, confirming the obtainment of the purified recombinant enzyme, and suggest the presence of the protein in bands corresponding to aggregates that could be dimers. The presence of faint bands with intermediate molecular weights may also indicate the existence of intermediate forms, probably due to the sensitivity of LiUBC1 to degradation agents even in the presence of protease inhibitors.

### 3.4. LiUBC1 Expression Levels throughout the Growth Curve of IPER/ES/2013/ATE1FL6 Strain of L. infantum Promastigotes in Axenic Culture

**Figure 4:**
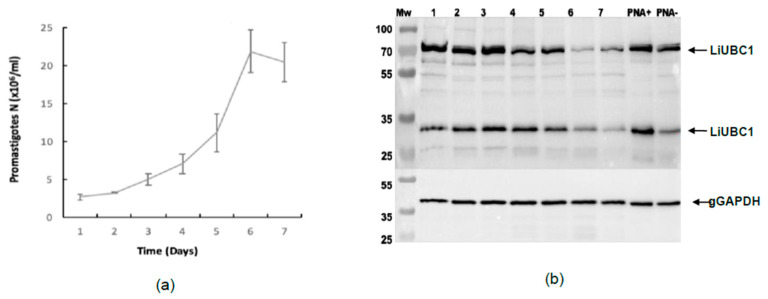
LiUBC1 expression patterns: (**a**) growth curve of the human outbreak strain (IPER/ES/2013/ATE1FL6); (**b**) Western blot analysis of the LiUBC1 protein extracted from parasite samples taken during the different days of growth of the human outbreak strain and the samples obtained with the PNA selection. Arrows indicate the possible weight corresponding to the monomer (~35 kDa) and dimer (~70 kDa) bands of the protein. All densitometry data of each band can be seen at Supplementary Materials file (Figure S2).

We studied the expression of LiUBC1 in the highly infective IPER/ES/2013/ATE1FL6 strain obtained from *P. perniciosus* gut during a human outbreak of the disease in Spain (Section 2.1). Figure 4 shows the growth curve, as well as the expression of the LiUBC1 protein. The strain from the human outbreak (Figure 4A) has a lag phase that extends to day three, followed by a sharp logarithmic phase that reaches a maximum at day 6 and a short stationary phase before the rapid start of cell death on day 7. 

The Western blot analysis of the extracts obtained from each day of culture also showed the different protein expression patterns of LiUBC1. The strain from the human outbreak displayed two bands that may correspond to the monomer and dimer forms of the enzyme, with a slight decrease at the stationary phase of growth as indicated by the gGADPH controls, as well as the presence of faint intermediate forms. 

The LiUBC1 expression pattern was similar in the PNA selection extracts, though there was a lower expression of the monomeric form in the PNA extracts. 

### 3.5. Subcellular Localization of LiUBC1 in the L. infantum Parasite

**Figure 5:**
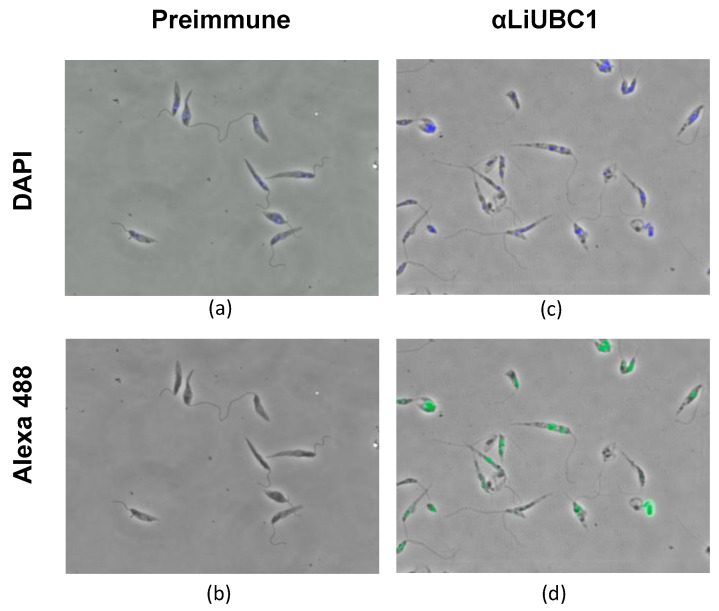
Indirect immunofluorescence of *L. infantum* (IPER/2013/ATE1FL6) promastigotes. The rabbit preimmune serum (**a**,**b**) and the anti-LiUBC1 polyclonal antibody (**c**,**d**) were used. The secondary antibody was the goat antirabbit IgG conjugated to Alexa Fluor 488. As can be seen in the figure, the LiUBC1 protein is localized in the nucleus and the kinetoplast area (green fluorescence from Alexa Fluor 488 (**b**,**d**) colocalized with DAPI (**a**,**c**) in blue).

IIF experiments with the anti-LiUBC1 polyclonal antibody displayed a particulate distribution of the protein in two parasite areas: the nucleus and the kinetoplast area (Figure 5). This technique did not allow us to differentiate between the nuclear membrane and the perinuclear cytoplasmic zone. The same happened with the presence of the protein in the flagellar pocket area. To clarify this point, we carried out TEM experiments. 

**Figure 6:**
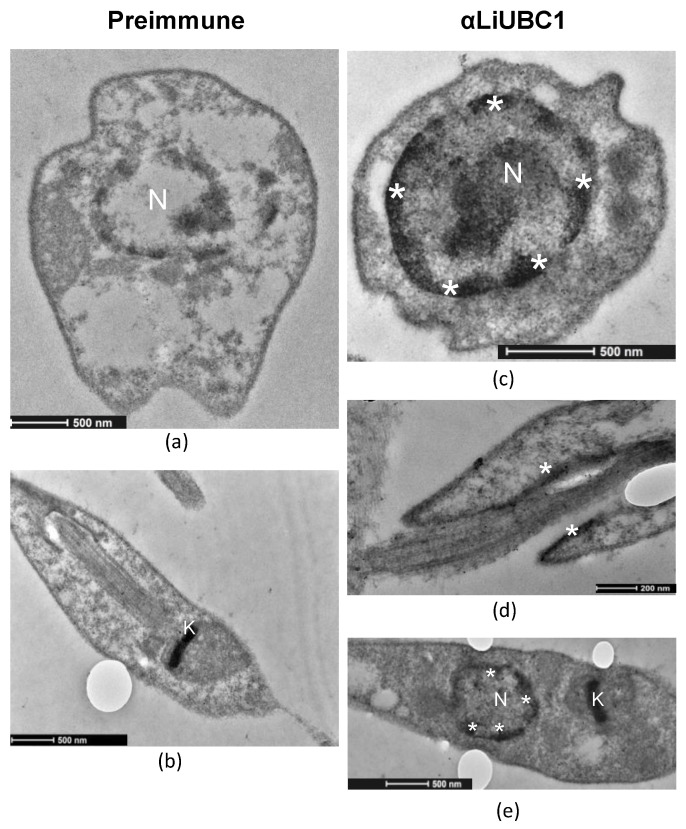
Immunolocalization of LiUBC1 using TEM. Asterisks (*) indicate the presence of gold precipitate corresponding to the presence of LiUBC1 protein in the different parasite subcellular structures. (**a**) Transverse section of *L. infantum* promastigotes incubated with preimmnune antibody. (**b**) *L. infantum* promastigotes incubated with preimmnune antibody. (**c**) Transverse section of *L. infantum* cell incubated with anti-LiUBC1 antibody. (**d**) Detail of the flagellar pocket area incubated with specific anti-LiUBC1 antibody. (**e**) Detail of the parasite’s nucleus incubated with specific serum anti-LiUBC1 antibody. N—nucleus; K—kinetoplast.

TEM using the same LiUBC1 primary antibody and the antirabbit IgG conjugated to colloidal gold was carried out to obtain more accurate information about the LiUBC1 subcellular localization. This experiment revealed that the protein is located in the nuclear envelope area, as well as in the kinetoplast and adjacent to the flagellar pocket membrane (Figure 6).

The TEM experiments confirmed the presence of LiUBC1 in the nuclear membrane area, as well as the flagellar pocket and the kinetoplast.

### 3.6. Characterization of an L. infantum Stable LiUBC1 Knock-In Promastigote Cell Line

**Figure 7:**
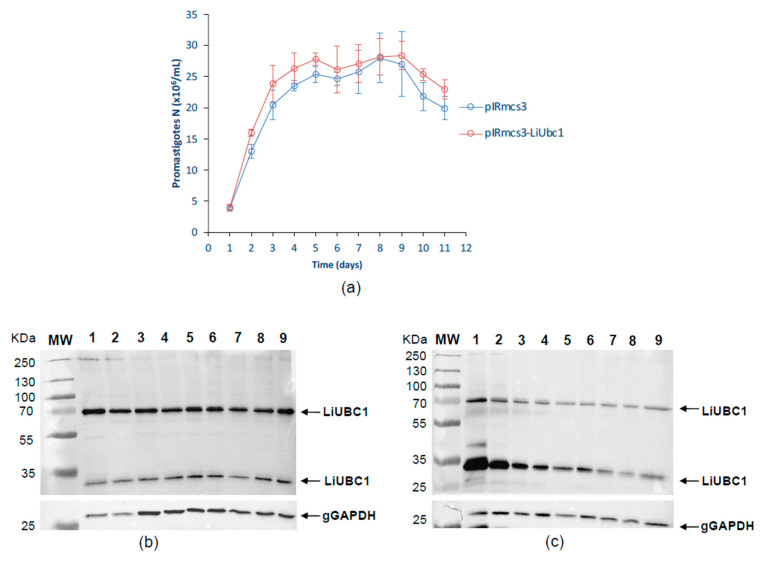
Growth and LiUBC1 levels in the pIRmcs3-LiUBC1 knock-in *L. infantum* promastigote cell line: (**a**) growth curve of the transfected *L. infantum* strain, transfectants containing the pIRmcs3 vector in blue and pIRmcs3-LiUBC1 knock-in cell line in red; (**b**) Western blot pattern of the LiUBC1 protein throughout growth of pIRmcs3 control transfectants; (**c**) pIRmcs3-LiUBC1 knock-in promastigotes.

A knock-in cell line was generated for functional studies using the infective human outbreak strain (Section 2.1). Figure 7A shows the growth pattern of *L. infantum* parasites overexpressing LiUBC1. Both growth curves, corresponding to the pIRmcs3-LiUBC1- and the pIRmcs3-transfected promastigotes, show similar patterns. The expression levels of the LiUBC1 protein are similar throughout the growth curve in the case of the pIRmcs3-transfected promastigotes (Figure 7B). The transfected promastigotes overexpressing LiUBC1 showed a higher expression of LiUBC1 at the beginning of the growth curve, mostly in the first 24 h (Figure 7C).

We validated the results shown in Figure 7 using iTRAQ. We analyzed the proteome pattern of the transfected parasite and the possible modifications induced by this noticeable increase in the ubiquitin-conjugating E2 enzyme on other related enzymes of the ubiquitin-conjugating system. Thus, we used the following ubiquitin enzymes (putative) as a reference: the E1-activating E1 enzyme, the ubiquitin-conjugating E2 enzyme, and the ubiquitin-conjugating E3 enzyme, corresponding to the LinJ.23.0710, LinJ.35.1310, and LinJ.13.1320 genes, respectively. Table 3 shows the levels of the relative abundance of these proteins with respect to LiUBC1. The overexpression of LiUBC1 does not affect the production of these similar conjugating and activating enzymes. Thus, proteins belonging to the ubiquitin proteasome system are not generally overexpressed, while those of ubiquitin modification systems are, specifically, LiUBC1. 

### 3.7. Infectivity of LiUBC1 Knock-In Promastigotes in U937 Cells In Vitro 

**Figure 8:**
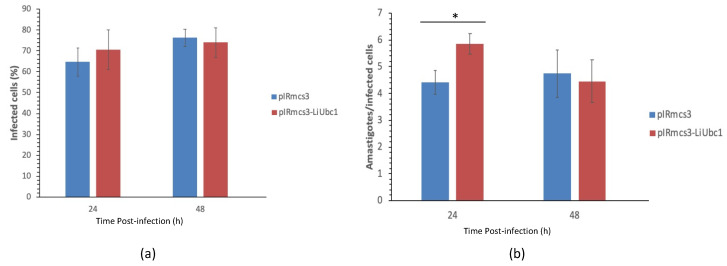
In vitro infection of U937 myelomonocytic cells with transfected *L. infantum* parasites: (**a**) percentage of infected phagocytes at 24 and 48h post-infection; (**b**) number of amastigotes per infected cell at 24 and 48h post-infection. Blue bars: infection with parasites transfected with empty plasmid. Red bars: U937 cells infected with the LiUBC1 overexpressing plasmid. *Statistical inference was performed using the Student’s t-test * *p* < 0.05.

In vitro infection experiments were performed using the PMA stimulated U937 cell line model. Figure 8 shows the percentage of U937 cells infected by *L. infantum* LiUBC1 knock-in promastigotes (red bars) and pIRmcs3 transfected promastigotes (blue bars). Slight differences are observed (Figure 8A). A statistically significant (*t*-test, *p* < 0.05) 27% average increase in the number of amastigotes per infected cell was observed in the 24 h post-infection timeframe (Figure 8B), i.e., early infection stage.

An increase of the presence of intracellular amastigotes in U937 cells infected with promastigotes overexpressing the LiUBC1 gene in the early phases (Figure 8) suggests a probable role of LiUBC1 in the parasite infection process. 

### 3.8. Protective Effect of the LiUBC1 Recombinant Protein in M. auratus against L. infantum Challenge

The possible role of the LiUBC1 recombinant protein as a protective molecule against *L. infantum* infection was evaluated herein. The investigation was performed using the *M. auratus* model in a challenge experiment with *L. infantum* promastigotes. Four months post-infection, 50% of the control animals showed the presence of *L. infantum* amastigotes in the spleen, whereas animals vaccinated with the recombinant LiUBC1 protein did not (Table 4).

**Figure 9:**
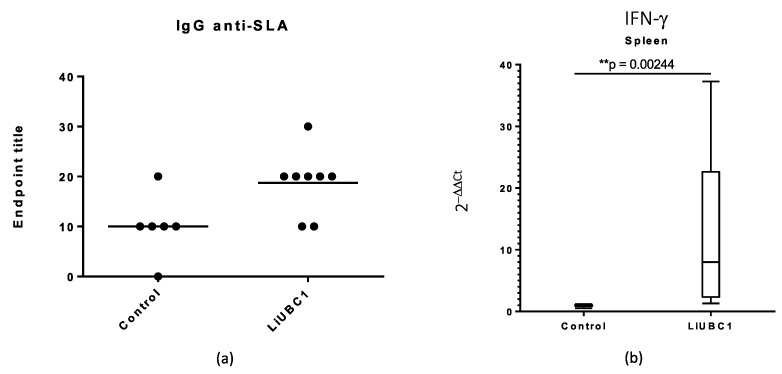
Evaluation of the specific immune response in *M. auratus*: (**a**) humoral immunity (IgG anti-SLA); (**b**) cellular immunity. We used qPCR for the quantification of the relative IFN-γ mRNA levels in splenocytes (IFN-γ). Statistical inference was performed using the Student’s t-test (** *p* < 0.01).

Additionally, we analyzed the IgG anti-SLA and IFN-γ production. As can be seen in Figure 9A, the production of IgG anti-SLA was increased in the animals inoculated with LiUBC1 recombinant protein. Additionally, the IFN-γ production in the spleen cells of the animals inoculated with the recombinant protein showed a statistically significant increase (Figure 9B).

The results of the parasite burden, the IgG anti-SLA, and the IFN-γ production suggest the possible role of the LiUBC1 recombinant protein as a protective molecule against *L. infantum* infection.

## 4. Discussion

LiUBC1 is a protein involved in degradation and regulation mechanisms essential for the adaptation of the cell to distinct physiological conditions, helping to maintain cell homeostasis. Processes such as DNA repair or protein synthesis are regulated by ubiquitin [34]. This may be relevant for *Leishmania* parasites, which have to adapt to the different environments along its life cycle in two different hosts, from the vector gut to the macrophage parasitophorous vacuole. We characterized the protein in a lower eukaryote, and it displays structural features similar to other members of the *Leishmania* genus and other lower eukaryotes, such as *S. cerevisiae*, but dissimilar to other members of the Trypanosomatidae family. The protein structure of LiUBC1, predicted based on the data obtained from *S. cerevisiae* and *H. sapiens* orthologs, showed a C-terminal end very similar to that observed in the yeast protein, which is not present in the human enzyme (Figure 2). This fact suggests that the LiUBC1 enzyme may be a good vaccine or drug target candidate, whose blockage through the C-terminus would only affect the parasite’s survival and not their host, providing a good pharmacological advantage for a possible treatment for humans infected with *L. infantum*. This increases the interest in unraveling the characterization of LiUBC1 from *L. infantum,* responsible for VL in the Mediterranean basin, China, and South America.

The purified recombinant LiUBC1 protein displayed a complex pattern with the presence of two main bands (Figure 3) that showed molecular weights corresponding to the monomer and the dimer, as confirmed by LC–MS/MS (Table 2). A specific antibody was obtained to study the LiUBC1 expression pattern and subcellular localization throughout the promastigote growth curve. 

The immunofluorescence experiments showed localization in the nuclear area and close to the base of the parasite’s flagellum. TEM analysis confirmed the presence of LiUBC1 in the nuclear membrane zone, as well as the kinetoplast and the flagellar pocket area. This localization corresponds to the assigned functions of the protein. The protein is only located near protein-synthesis and -modification areas, and not throughout the entire cytoplasm, in a parasite in which autophagy and endosome sorting are essential in metacyclogenesis [56]. In *Leishmania*, the perinuclear zone has been shown to control post-translational regulation [57], which would require the presence of ubiquitin activity. The internalization of exogenous polypeptides and proteins through the flagellar pocket (cytostome) may be related to ubiquitin targeting for degradation [37]. The generation of knock-in transfectants helps us study the possible relation of the ubiquitin-conjugating E2 protein with the parasite’s infectivity. LiUBC1-transfected promastigotes showed a clear predominance of the monomer form over the aggregate. The LiUBC1 knock-in promastigote line reached the highest levels of protein expression at the beginning of the growth curve (Figure 7). This high amount of the monomeric form rapidly decayed at 72 h, reaching normal levels. The iTRAQ-based proteome analysis of the LiUBC1 knock-in promastigotes revealed that LiUBC1 overexpression did not affect the intracellular levels of any other identified protein. Overexpression was confirmed in the pIRmcs3-LiUBC1 stable knock-in promastigote cell line. Growth and morphology were not altered in this cell line and, according to iTRAQ analysis, constitutively induced LiUBC1 expression did not lead to pleiotropic effects consisting of substantial changes in the proteome. The cell line overexpressing the LiUBC1 gene was more infective to PMA-stimulated U937 cells during the 24 h post-infection, as seen by the increase in intracellular amastigotes in the infected cells. The increase in infectivity at an early stage induced by LiUBC1 gene overexpression (Figure 8) suggests that immunization with this molecule may protect the host against infection. A protection experiment against a challenge with *L. infantum* in the *M. auratus* model was performed. In this experiment, the humoral and cellular immune response against the parasite was increased in the vaccinated animals. Furthermore, inoculation with the protein elicited a moderate, although clear, protection against parasite infection. These results agree with those obtained using a closely related E2 enzyme from *L. donovani,* which showed its ability to protect against infection in the mouse model [58]. Recent data indicate the presence of specific antibodies against the E2 protein in human serum obtained from VL patients [59].

Taken as a whole, these results are encouraging and indicate that further studies should be carried out on the role of the ubiquitin-conjugating E2 enzyme in the key functions of *L. infantum* metabolism, especially in the regulation processes. The unraveling of the role of the enzyme as a response to the parasite’s environmental conditions along its biological cycle would provide basic knowledge on the regulation mechanisms of this protozoa. This knowledge would also be useful for the development of effective drugs or vaccines against the parasite.

## 5. Conclusions

LiUBC1 is similar (>80%) to orthologs from the *Leishmania* spp., but not to the mammalian orthologs included in the comparison (<40%), which lack the C-terminal domain found in *Leishmania* spp. According to the Western blot experiment, the LiUBC1 gene is expressed throughout *L. infantum* promastigote growth, including highly infective PNA- promastigotes. The Syrian hamster protection experiment against challenge with *L. infantum* revealed a partial reduction in parasite burden and IFN-γ mRNA levels in the spleen. Therefore, LiUBC1 is a vaccine candidate that confers partial protection in Syrian hamsters against *L. infantum* infection.

## Figures and Tables

**Table 1:** Percentages of identity between different LiUBC1 orthologs.

Species	% Identity
*Leishmania donovani*	100
*Leishmania major* *Leishmania mexicana* *Leishmania braziliensis* *Trypanosoma cruzi* *Oryctolagus cuniculus* *Homo sapiens* *Xenopus laevis* *Arabidopsis thaliana* *Drosophila melanogaster* *Mesocricetus auratus*	100 91.70 80.50 48.98 38.09 31.30 31.30 30.82 27.14 20.17

**Table 2:** LiUBC1 protein band identification by nLC–MS/MS. The protein bands were excised from Table 3. C. Proteins were extracted from the gel and analyzed by nLC–MS/MS. The MASCOT score is a statistical parameter that indicates that the identification is reliable (*p* < 0.05) when values are higher than 52. The coverage indicates the percentage of the protein sequence which has been detected in each case.

BAND	Gene	Description	Score	Coverage
**1**	LinJ.33.2910	Ubiquitin-conjugating enzyme E2	1235	91.70
**2**	LinJ.33.2910	Ubiquitin-conjugating enzyme E2	487	70.54

**Table 3:** Results of the LC–MS/MS analysis (iTRAQ). The expression levels of the LiUBC1 protein and those of other genes related to the ubiquitins of *L. infantum* are shown. The minimum level of relative abundance was set at a ratio of 2 (log2 ratio ≥ 1).

Gene	Description	Mean pIRmcs3-LiUBC1 vs. pIRmcs3		
		Day 1	Day 3	Day 5
LinJ.33.2910	LiUBC1 Ubiquitin-conjugating enzyme E2, putative	2.759 ± 0.30	0.031 ± 0.11	0.327 ± 0.51
LinJ.23.0710	Ubiquitin-conjugating enzyme E1, putative	0.008 ± 0.01	0.062 ± 0.07	0.083 ± 0.06
LinJ.35.1310	Ubiquitin-conjugating enzyme E2, putative	0.021 ± 0.01	0.095 ± 0.14	0.264 ± 0.25
LinJ.13.1320	Ubiquitin-conjugating enzyme, putative	0.225 ± 0.29	0.317 ± 0,40	0.087 ± 0.59

**Table 4:** Evaluation of the parasite load by limiting dilution in the protection experiment against *L. infantum* infection of hamsters inoculated with the LiUBC1 recombinant protein. Four technical replicates of serial dilutions were made. The geometric mean of the dilution limit (M_G_) was used to calculate the number of amastigotes per gram of tissue.

Group	Sample Nº	Spleen Weight (g)	M_G_ Dilution Limit	Amas/g Spleen
**LiUBC1**	1 2 3 4 5 6 7 8	0.1123 0.1099 0.0844 0.1236 0.1025 0.0844 0.0925 0.1299	- - - - - - - -	- - - - - - - -
**Control**	9 10 11 12 13 14	0.1371 0.0972 0.1161 0.1171 0.0752 0.1738	- 16 - - 32 256	- 2469 - - 6382 22094

## Data Availability

All data are included in the manuscript and the supplementary files.

## Supplementary Materials

The following supporting information can be downloaded at: https://www.mdpi.com/article/10.3390/vaccines10020231/s1, Figure S1: Comparative alignment of the primary structure of the LiUBC1 protein, Figure S2: Raw Western Blot Images.
