# Supplementary material for: Leishmania infantum UBC1 in Metacyclic Promastigotes from Phlebotomus perniciosus, a Vaccine Candidate for Zoonotic Visceral Leishmaniasis
Source: Vaccines (Basel). 2022 Feb 3;10(2):231. doi: 10.3390/vaccines10020231 (PMC8877641; doi:10.3390/vaccines10020231)
Supplement: Supplementary file 1 [file vaccines-10-00231-s001.zip › vaccines-1531710-supplementary.pdf]

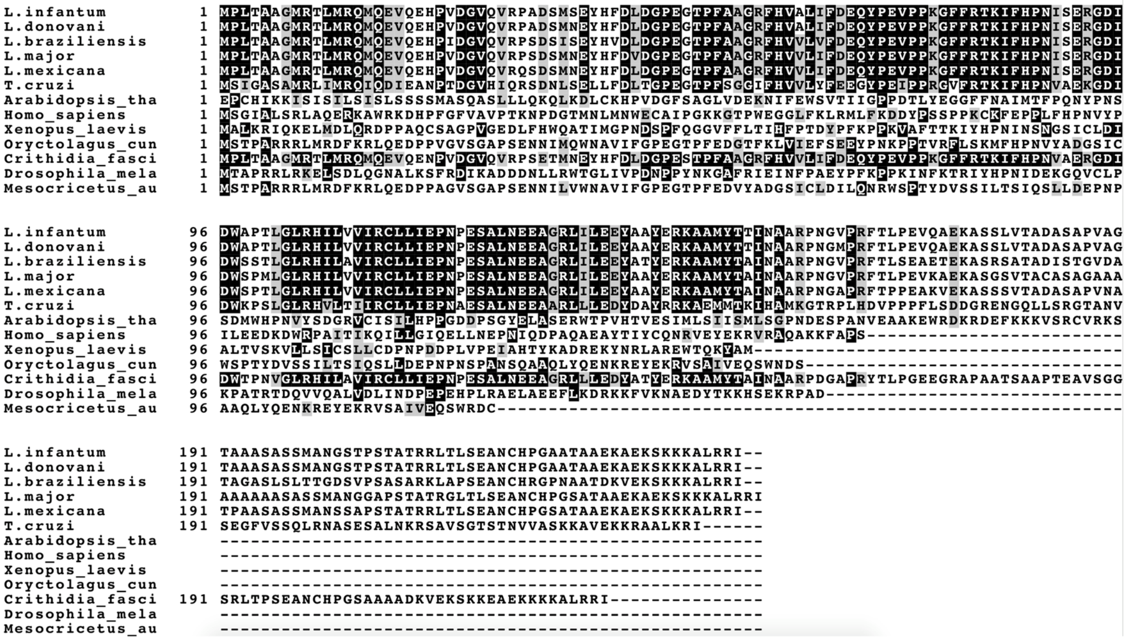

Figure S1. Comparative alignment of the primary structure of the LiUBC1 protein. The degree of conservation is represented in gray scale both in species of the trypanosomatid family and in different eukaryotes.

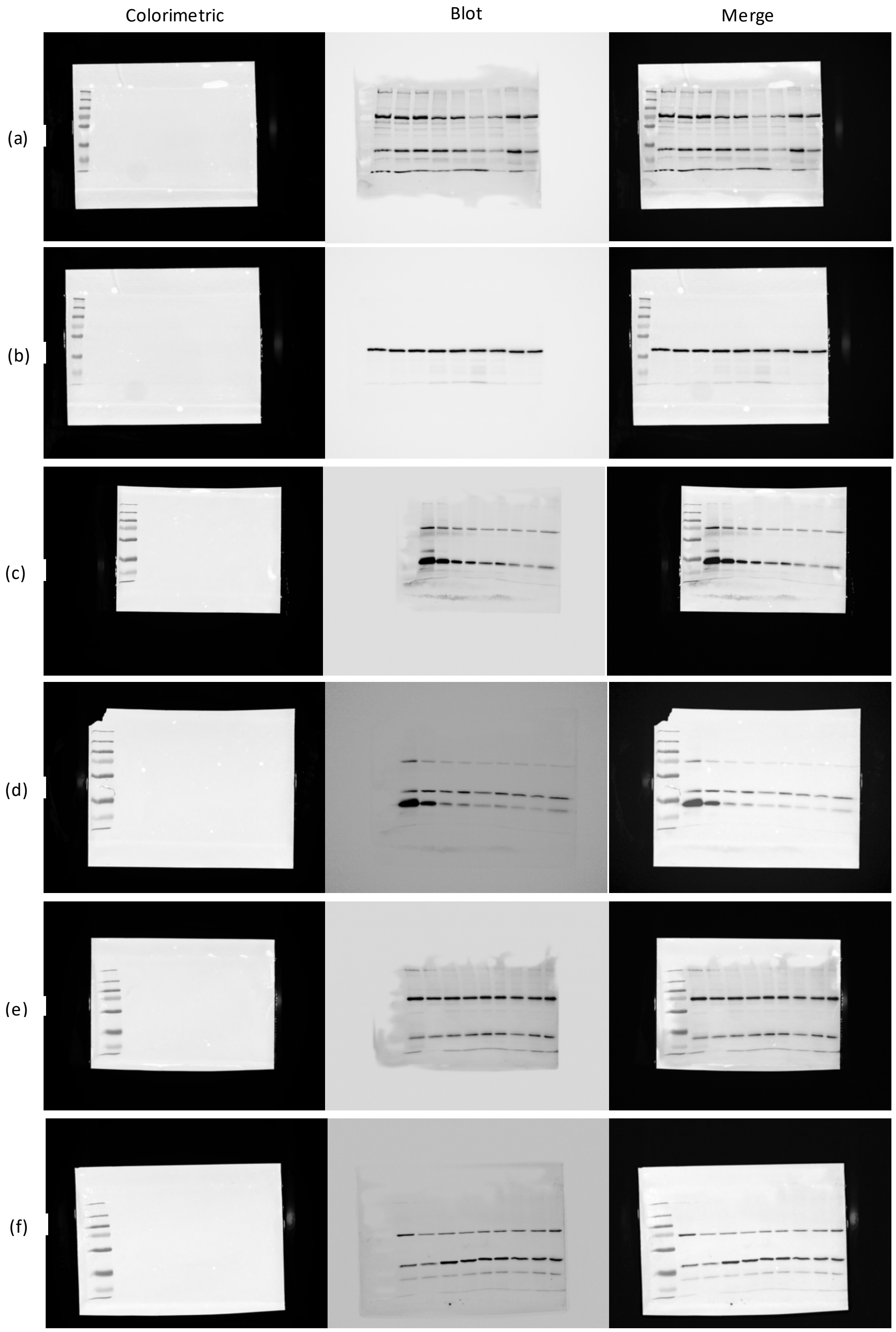

Figure S2. Raw Western Blot Images. Densitometry readings are in supplementary materials. **(a)** LiUBC1 expression pattern corresponding Figure 4(b) **(b)** LiUBC1 expression pattern antigGAPDH corresponding Figure 4(b) **(c)** Western blot pattern of the LiUBC1 protein throughout growth of pIRmcs3 control transfectants corresponding to Figure 7(b) **(d)** Western blot pattern of the LiUBC1 protein throughout growth of pIRmcs3 control transfectants antigGAPDH corresponding to Figure 7(b) **(e)** pIRmcs3-LiUBC1 knock-in promastigotes corresponding to Figure 7(c) **(f)** pIRmcs3-LiUBC1 knock-in promastigotes antigGAPDH corresponding to Figure 7(c).
